# Enigmatic tracks of solitary sauropods roaming an extensive lacustrine megatracksite in Iberia

**DOI:** 10.1038/s41598-021-95675-3

**Published:** 2021-08-20

**Authors:** Fidel Torcida Fernández-Baldor, I. Díaz-Martínez, P. Huerta, D. Montero Huerta, D. Castanera

**Affiliations:** 1 Museo de Dinosaurios de Salas de los Infantes and Colectivo Arqueológico y Paleontológico de Salas (CAS), Plaza Jesús Aparicio 9, 09600 Salas de los Infantes, Burgos Spain; 2 Universidad Nacional de Río Negro. Instituto de Investigación en Paleobiología y Geología, General Roca, Río Negro Argentina; 3 Instituto de Investigación en Paleobiología y Geología (IIPG), CONICET, Av. Roca 1242, 8332 General Roca, Río Negro Argentina; 4 Dpto. Geología, Escuela Politécnica Superior de Ávila, Universidad de Salamanca, Avda. Hornos Caleros, nº 50, 05003 Ávila, Spain; 5 Institut Català de Paleontologia Miquel Crusafont, Universitat Autònoma de Barcelona, c/Escola Industrial 23, 08201 Sabadell, Barcelona Spain

**Keywords:** Evolution, Palaeontology, Palaeontology, Sedimentology, Geology, Stratigraphy, Limnology

## Abstract

Sauropod remains are abundant on the Iberian Peninsula across the Jurassic-Cretaceous transition. Where the osteological record shows a high diversity of this kind of dinosaur, the ichnological findings are mainly limited to sauropod tracks characterized by kidney-shaped manus (with or without pollex impressions) and pes impressions with three claw imprints oriented laterally. Here, we present a new sauropod ichnotaxon, *Iniestapodus burgensis*, found at several exposures within the Las Sereas megatracksite (Burgos, Spain). These are preserved within lacustrine limestone strata of the Rupelo Formation (Tithonian–Berriasian). *Iniestapodus burgensis* is characterized by: semicircular manus tracks with small pollex impressions; unusual tetradactyl pes tracks with evidence of four claws oriented anteriorly (I–II) and laterally (III–IV), of variable sizes (short claw I and IV impressions, claw II and III being the largest). The combination of features and comparison with the osteological record allows us to propose a non-titanosaurian titanosauriform as a possible trackmaker. All the *Iniestapodus* tracks are represented by at least two different size classes of small and medium-sized individuals, and their trackways show different multidirectional orientations. The paleoenvironmental and paleoecological data suggest that *Iniestapodus* trackmakers were solitary individuals, likely representing different age classes, that crossed and used the Las Sereas shallow lacustrine-palustrine areas as their preferred habitat.

## Introduction

The Late Jurassic (Kimmeridgian-Tithonian) and Jurassic-Cretaceous (Tithonian–Berriasian) intervals constitute time periods in which most Iberian Peninsula sauropod tracks have been described, with significant sites in the Lusitanian, Asturian, Cameros, and Maestrazgo basins. Generally, these track impressions are characterized by a kidney-shaped manus (either with or without a claw impression on digit I) and subtriangular pes with claw impressions generally oriented anterolaterally, and have been associated with the *Parabrontopodus*-like/*Breviparopus*-like and *Brontopodus*-like ichnotypes^1,2^.

The sauropod osteological record, although from fewer sites, has yielded a large number of remains derived mainly from the Lusitanian basin^3^ and the Iberian Basin Rift System^4,5^. This record includes taxa ranging from basal Macronaria (*Lourinhasaurus alenquerensis*^6^ and *Aragosaurus ischiaticus*^7^), Titanosauriformes (*Lusotitan atalaiensis*^8^, *Galvesaurus herreroi*^9^ and *Oceanotitan dantasi*^10^), Diplodocoidea (*Dinheirosaurus lourinhanensis*^11^) to Turiasauria (*Losillasaurus giganteus*^12^, *Turiasaurus riodevensis*^4^ and *Zby atlanticus*^13^), as well as material not assigned to a specific taxon but representing the aforementioned four clades^3,5,14,15^.

In the Iberian context, the Tithonian–Berriasian Rupelo Formation (Tera Group, Cameros Basin) preserves, remarkably, both sauropod bones and tracks^15–18^. Recently, a humerus attributed to a basal brachiosaurid has been described^15^. In general, the sauropod tracks are preserved at the top of a carbonate level that is exposed over an extensive area with ~ 14 track-bearing outcrops along 5.6 km^17^. This extensive tracksite, which constitutes a megatracksite^19^, is called Las Sereas and preserves more than 1000 dinosaur footprints^17^. Theropod, ornithopod, thyreophoran, and sauropod tracks have been found, with the latter group being the most abundant^17,18^. Moreover, a singular sauropod dinosaur ichnotype, characterised by pes tracks with four claw impressions, was described at one outcrop, Las Sereas 7 (LS7)^18^, within the Las Sereas megatracksite. Recently, new sauropod tracks and trackways with the same characteristics as this ichnotype, but of two different size classes, have been identified in two further Las Sereas outcrops. These recent discoveries expand the distribution and current information on these specific tracks and their trackmakers.

This study has multiple objectives. Firstly, the aims are to describe the new sauropod tracks, to compare them with tracks of the LS7 outcrop, and to analyze their ichnotaxonomic affinities. Secondly, herein we discuss the taxonomic relationships of the producers of these tracks. Finally, we infer the palaeoenvironmental and palaeoecological significance of the presence of these sauropod tracks in the lacustrine megatracksite of Las Sereas.

## Geological setting

**Figure 1:**
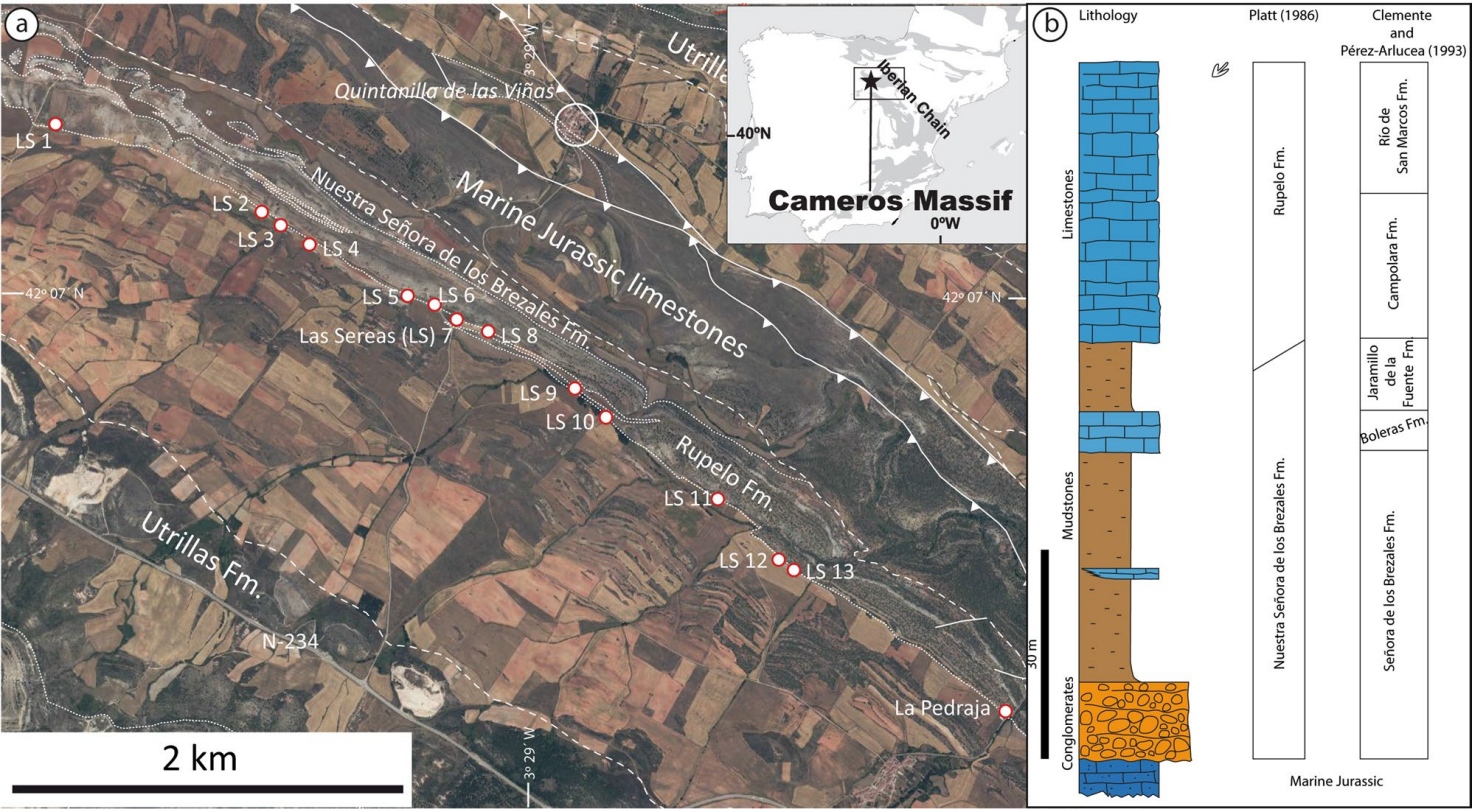
Geological and geographical setting of the Las Sereas tracksite. (**a**) Geological sketch showing the different tracksites at Las Sereas (LS 1—LS 13, and La Predraja). The map has been generated from fieldwork and mapped on the orthophoto (PNOA, www.ign.es) with Arcgis 10.3. (https://desktop.arcgis.com) (**b**) Schematic geological section from Quintanilla de las Viñas showing the equivalence of the units proposed by Platt^23^ and those from Clemente and Perez-Arlucea^25^. The figure drawings were made with Adobe Illustrator CS2; the geological map, site locations, and the stratigraphic section were created by one of the authors (P. Huerta) of this manuscript.

The Las Sereas tracksite is located 1.2 km SW of Quintanilla de las Viñas (SE of the Burgos province) and 10 m apart from the road BU-V-8207 (Fig. 1a). The sedimentary succession belongs to the western part of the Cameros Basin, which is located in the northwestern domain of the Iberian Basin Rift System that was developed during the Late Jurassic–Early Cretaceous^20–22^. The Las Sereas tracksite occurs at the top of the Rupelo Formation^23,24^. The upper part of the Rupelo Formation is equivalent, following other stratigraphic frameworks, to the Río de San Marcos Formation (depositional sequence 3)^25,26^ (Fig. 1b). The biostratigraphy of this succession is based on charophytes and ostracods and suggests a Tithonian–Berriasian age for the Rupelo Formation and equivalent lithostratigraphic units^26,27^. The upper part of the Rupelo Formation extends into the early to middle Berriasian^22,24,28^ based on the occurrence of *Globator maillardii* var. *incrassatus* in the Mambrillas section^29^ and on the Berriasian ostracod assemblage found in the underlying Campolara Formation^27^, which is equivalent to the middle part of the Rupelo Formation. These stratigraphic associations imply that the Las Sereas tracksite is Berriasian in age.

The Rupelo Formation comprises lacustrine and palustrine limestones with charophytes, ostracods, gastropods, dinosaur bones and tracks that have been interpreted as having been deposited on the low-gradient margin of a carbonate precipitating lake with seasonal lake level fluctuations and pedogenesis^30^. The occurrence of benthic foraminifers (miliolids) and dasycladales, in the upper part of the Rupelo Formation, is one of the key elements suggesting a new carbonate coastal wetland sedimentary model for the Río de San Marcos Formation^22^. The Las Sereas tracksite is located within the Quintanilla section, where little to no marine influence in the coastal wetlands is evident^22^.

The Las Sereas tracksite occurs in one of the last limestone beds within the uppermost Rupelo Formation. This limestone bed has wackestone to packstone textures that, in some parts, show exposure features such as peloidal-intraclastic textures, alveolar and channel porosities, and vadose silt in vug or crack porosities. At the top of the bed, some circular impressions with diameters of 0.4–0.8 cm and lengths of 4–5 cm when are observed in section, are interpreted as marks of plant stems and/or roots.

## Material and methods

In this work, the best-preserved quadrupedal tracks found in the Las Sereas 3 (LS3), Las Sereas 7 (LS7), and Las Sereas 8 (LS8) tracksites are documented and described. All the tracks are in situ and were documented using 2D cartography and photogrammetry of the most representative tracks in addition to a laser scanner model of part of the LS8 surface.

At the LS8 site, about 140 dinosaur tracks were identified, although the track-bearing level still needs additional fieldwork to be completely cleaned. However, trackway LS8A and two consecutive manus-pes sets have already been cleaned and were studied. The tracks were labelled according to previous conventions^31,32^, as follows: first, the tracksite identification (e.g., LS8); second, the trackway (e.g., A); third, separated by a comma, the footprint (e.g., ‘3’); and fourth pes or manus abbreviations (e.g., p or m). For instance, LS8A, 2 m is the manus impression of the second manus-pes set of trackway A of tracksite LS8 (Las Sereas 8). LS8A is composed of 46 quadrupedal tracks (Table 1), whereas LS8B consists of two consecutive manus-pes sets: LS8B,1p/m and LS8B,2p/m.

**Table 1:** List of the tracks that belong to each studied trackway from the Las Sereas.

Trackway	Track labels
LS7A	LS7A,1p/m; LS7A,2p/m; LS7A,3p/m; LS7A,4p/m; LS7A,5p/m; LS7A,6p/m; LS7A,7p/m; LS7A,8p/m; LS7A,9p/m; LS7A,10p/m
LS7B	LS7B,1p/m; LS7B,2p/m; LS7B,3p/m; LS7B,4p/m; LS7B,5p; LS7B,6p; LS7B,7p
LS7C	LS7C,1p/m; LS7C,2p/m, LS7C,3m and LS7C,4p/m
LS8A	LS8A,1p/m; LS8A,2p/m; LS8A,3p/m; LS8A,4p/m; LS8A,5p/m; LS8A,6p; LS8A,7p/m; LS8A,8p/m; LS8A,9p/m; LS8A,11p/m; LS8A,12p/m; LS8A,13p/m; LS8A,14p/m; LS8A,15p/m; LS8A,16p; LS8A,19p/m; LS8A,20p/m; LS8A,21p/m; LS8A,22p/m; LS8A,23p/m; LS8A,24p/m; LS8A,25p/m; LS8A,26p/m; LS8A,27p/m
LS8B	LS8B,1p/m; LS8B,2p/m

LS3 represents ~ 400 tracks on a heavily trampled surface^33^. Only three of the best-preserved tracks were selected for this study, one isolated pes track and one manus-pes set of tracks labeled as LS3,1 and LS3A,1p/m, respectively.

Tracks belonging to three trackways, LS7A, LS7B, and LS7C (Table 1), previously documented and classified as aff. *Polyonyx*^18^, are also included. Some tracks of the LS7C trackway have been redrawn and remeasured, according to new available data (photogrammetry), showing some differences with respect to the previous work^18^.

The LS7 and LS8 tracksites represent the same track-bearing surface and are separated by only 160 m distance (see Supplementary Fig. S1). The LS3 tracksite is more than 950 m away from LS7 and LS8, and the tracks are impressed in the same set of strata but on a different layer. The surfaces of the LS7 and LS8 tracksites were divided into 1 × 1 m squares and each square was provided with a letter and a number to locate tracks with x- and y-coordinates. Field photos in LS3, LS7, and LS8 were taken with a Canon Power Shot S5IS camera (focal length 35 mm, resolution 3264 × 2448). Photogrammetry was undertaken following the general methodology explained in Falkingham^34^, Mallison and Wings^35^, and Falkingham et al.^36^. Generation of a three-dimensional, textured mesh was obtained running the process through the software Agisoft Metashape Pro (version 1.5.2, Educational License, http://www.agisoft.ru/) for a minimum of 10 to a maximum of 30 photos per track. Photogrammetric models in .obj format were also imported into CloudCompare (https://www.danielgm.net/cc/) to generate false-colour depth maps. The LS8A trackway was digitally acquired via the terrestrial laser scanner Faro Focus 3D X330 from the CENIEH (Centro Nacional de Investigación sobre la Evolución Humana, Burgos, Spain), with a sampling resolution of 2 mm, data collection speed of 976,000 points/s, and colour camera up to 70 mpx.

The terminology used in this paper mainly follows the works of Leonardi^37^, Farlow et al.^38^, and Thulborn^39^. The parameters were measured in the field and the laboratory and then verified using the 3D models. Several measurements were made (Supplementary Tables S1, S2): track length (L), track width (W), digit length (DLI, DLII, DLIII, DLIV), track rotation (FR), pace length (PL), stride length (SL), inner and outer trackway width (iTW–eTW), pace angulation (ANG), manus–pes distance (Dm–p), pes and manus width of the angulation pattern (WAP and WAM), and glenoacetabular distance (Gda) *sensu* Leonardi^37^. The heteropody was calculated with the heteropody index (HI) using the manus–pes area ratio (H) of Lockley et al.^40^ and the formula of González Riga and Calvo^41^: HI = [(Lm × Wm)/(Lp × Wp)]*100. To quantify the trackway gauge, the pes and manus trackway ratio (PTR and MTR) was calculated as TR = (pesW/eTW)*100^42^, and the WAP/PL ratio^43^ also was obtained. All parameters are given and compared in centimeters (cm), except FR and ANG (in degrees), H (adimensional), HI and PTR, and MTR (in %). Morphological quality (MP) has been analyzed using the scale proposed by Marchetti et al.^44^. Size classes follow Marty^45^, as: minute, PL < 25 cm; small, 25 cm < PL < 50 cm; medium, 50 cm < PL < 75 cm; and large, PL > 75 cm. The ichnotaxonomic analysis is based on the recommendations and ichnotaxobases proposed by Lockley et al.^40^, Wright^46^, Castanera et al.^2,47^, Kim and Lockley^48^, Díaz-Martínez et al.^49^, and Marchetti et al.^44^. The possible trackmaker is discussed based on comparisons between the osteological descriptions of the main groups of sauropods, and other quadrupedal dinosaurs, and the ichnological features of the Las Sereas tracks. Moreover, the geographical and temporal correlations of both records are also discussed^50^.

### Nomenclatural acts

The electronic edition of this article conforms to the requirements of the amended International Code of Zoological Nomenclature, and hence the new names contained herein are available under that Code from the electronic edition of this article. This published work and the nomenclatural acts it contains have been registered in ZooBank, the online registration system for the ICZN. The ZooBank LSIDs (Life Science Identifiers) can be resolved and the associated information viewed through any standard web browser by appending the LSID to the prefix “http://zoobank.org/”. The LSID for the publication is: urn:lsid:zoobank.org:pub:AEBDF6D8-BEE9-498C-A895-CD1CFB3252BA. The electronic edition of this work was published in a journal with an ISSN, and has been archived and is available from the following digital repositories: PubMed Central, LOCKSS.

## Results and discussion

### Ichnotaxonomy

*Iniestapodus* ichnogenus nov.

urn:lsid:zoobank.org:act:1A792344-00DA-478E-B774-5C55D2902E6C

aff. *Polyonyx* Torcida Fernández-Baldor et al. (2015, Figs. 2, 3a–d, 4a–f, 5a,b)^18^

**Type ichnospecies:***Iniestapodus burgensis.* Monospecific ichnogenus.

urn:lsid:zoobank.org:act:DDEF1EFE-9364-4C5C-BE94-D79910B303E3

**Holotype:** As for ichnospecies.

**Paratypes:** As for ichnospecies.

**Type locality:** As for ichnospecies.

**Type horizon:** As for ichnospecies.

**Derivatio nominis:** This ichnogenus is dedicated to Andrés Iniesta, the Spanish footballer who scored the winning goal in the 2010 World Cup final; -*podus* means foot in Greek.

**Diagnosis:** As for ichnospecies.

urn:lsid:zoobank.org:act:DDEF1EFE-9364-4C5C-BE94-D79910B303E3

*Iniestapodus burgensis* ichnosp. nov.

**Holotype:** A complete manus-pes set catalogued as LS7B,3m, and LS7B,3p. They are in situ and there is a photogrammetric model of both tracks.

**Paratypes:** The rest of the tracks of LS7B trackway, and the tracks included in LS7A, LS7C, LS8A, LS8B, LS3A,1p/m, and LS3,1 (see “Material and methods” for a complete list of tracks).

**Type locality:** Quintanilla de las Viñas (Burgos Province, Spain). Coordinates: UTM 30N (ETRS89) X 459,677; Y 4,662,743.

**Type horizon:** Rupelo Formation (upper part), Tithonian–Berriasian interval^16^, equivalent to Río San Marcos Formation, Berriasian in age^22,26,27^.

**Derivatio nominis:** The ichnospecific epithet of “burgensis” means from Burgos, the Spanish province where the tracks were found.

**Diagnosis:** Manus tracks roughly symmetrical, wider than long, and have a semicircular shape. In the best-preserved specimens, three short, broad, and rounded digit impressions (II, III, and IV) are anteriorly oriented, and a short and a subcircular digit I impression (pollex) is medially located. Pes tracks are longer than wide, with a subrectangular shape and four claw impressions. Claw I impression is smaller and more backwardly located than the others. Claw I and II impressions are forwardly oriented and impressions III and IV are slightly rotated and curved laterally. The lengths of the pes tracks are more than double that of manus tracks. The widths of the manus tracks are slightly smaller than the pes tracks.

### Description

**Figure 2:**
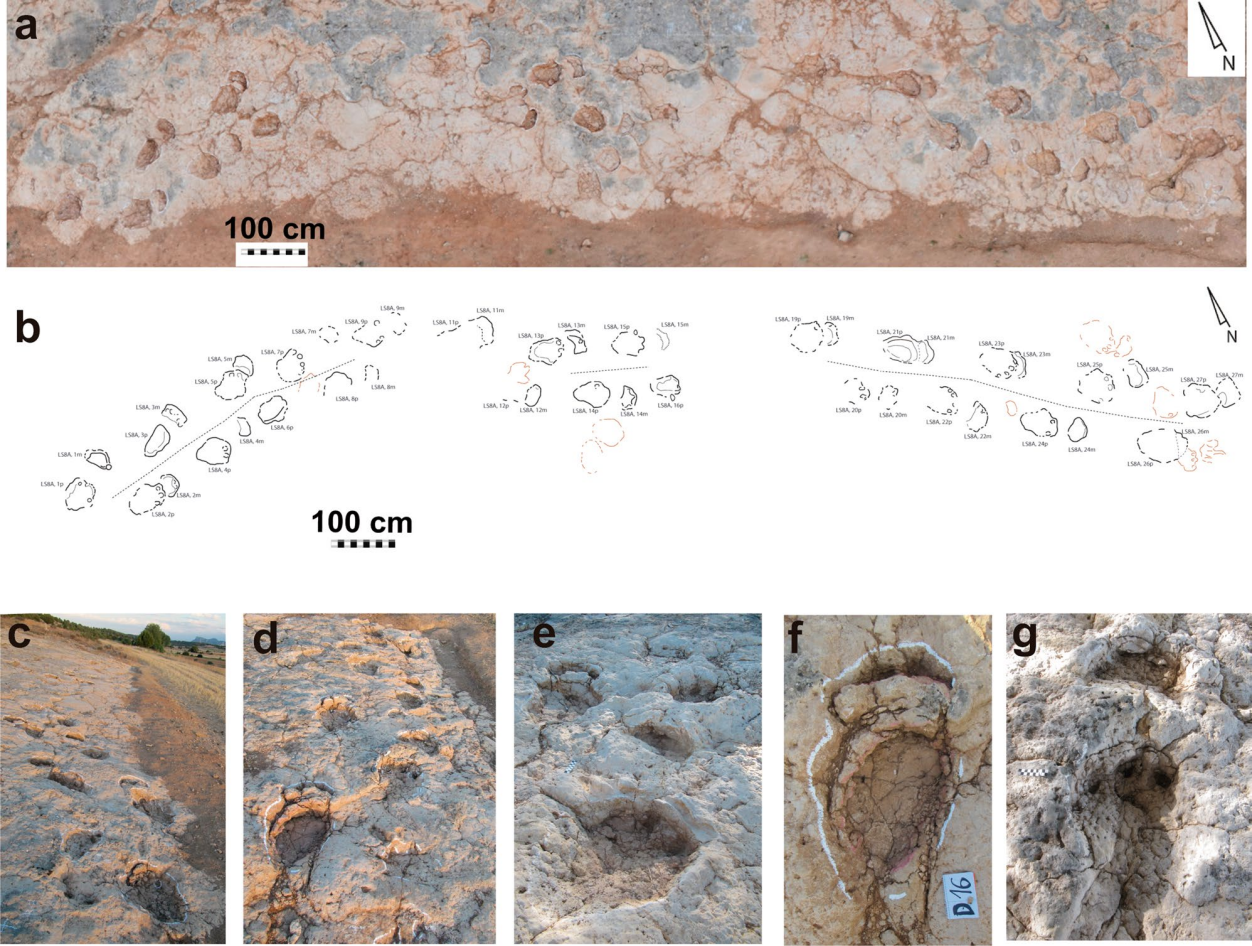
Las Sereas 8 tracksite. Tracks of *Iniestapodus burgensis*: (**a**) Ortophoto of LS8A trackway; (**b**) Interpretative cartography of LS8A trackway; (**c**) Initial section of LS8A trackway, from LS8A,1p to LS8A,7p; (**d**) Final section of LS8A trackway, from LS8A,20m to LS8A,27m; (**e**) A detail of the tracks LS8A,4p/ m; LS8A,5p/m; LS8A,6p; and LS8A,7p; (**f**) Pair LS8A,21p/m; (**g**) Pair LS8A,22p/m.

**Figure 3:**
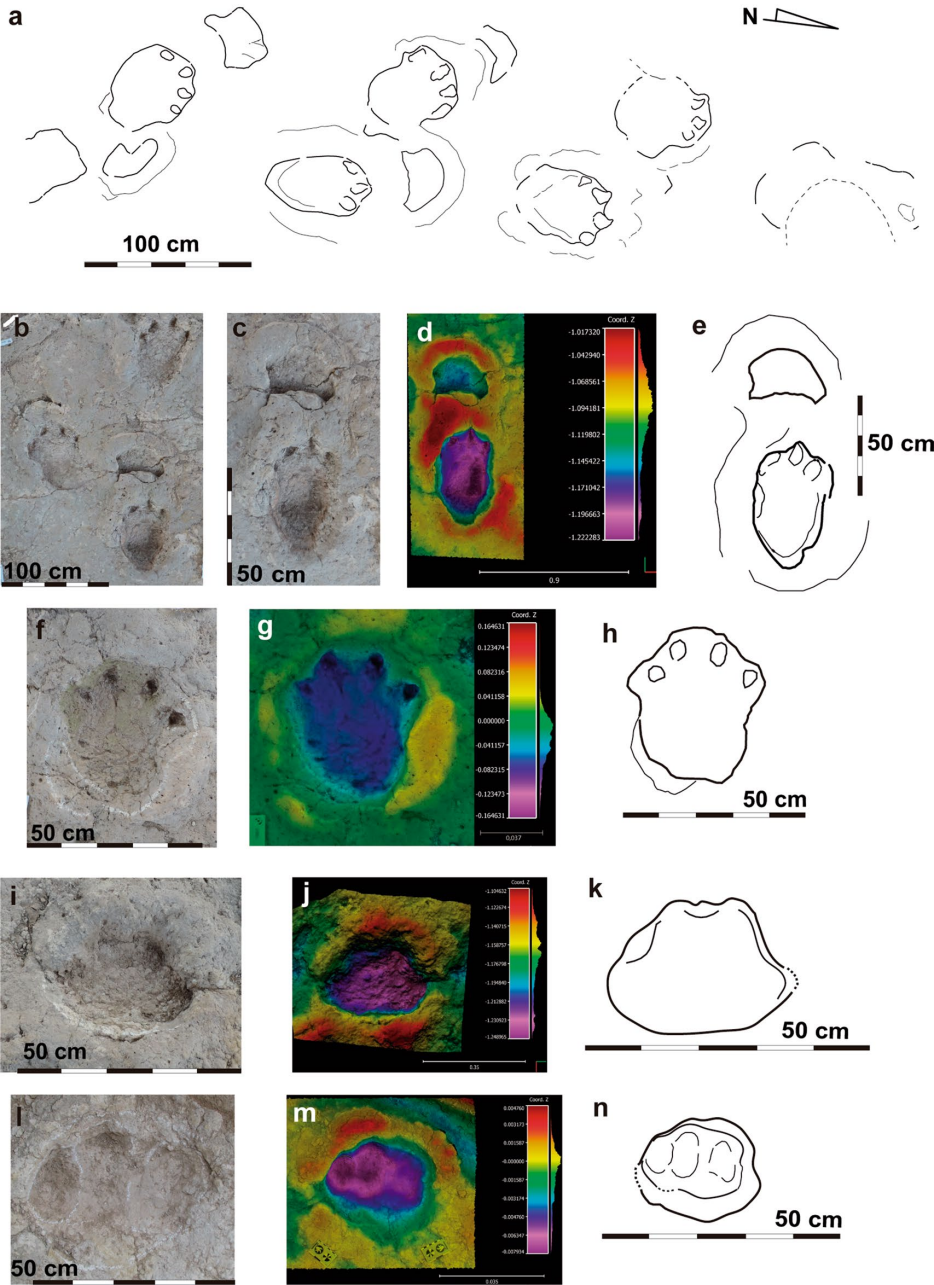
Las Sereas 7 tracksite. Holotype of *Iniestapodus burgensis*: (**a**) Interpretative cartography of LS7B trackway, modified from Torcida Fernández-Baldor et al.^15^. (**b**) Detail of the manus-pes sets LS7B,3p/m, 4p/m, and 5p. (**c-e**) Holotype LS7B,3p/m. (**c**) orthophoto; (**d**) false-color-depth map; (**e**) interpretative sketch. (**f-h**) Track LS7A,7p, right pes: (**f**) orthophoto; (**g**) false-color depth map; (**h**) interpretative sketch. (**i-k**) LS7A,8 m, left manus: (**i**) photography; (**j**) false-color depth map; (**k**) interpretative sketch. (**i-n**) LS7A,10m, right manus: (**l**) photography; (**m**) false*-color depth map; (**n**) interpretative sketch. The false-color depth map were generated with CloudCompare, a free software (http://www.cloudcompare.org/).

**Figure 4:**
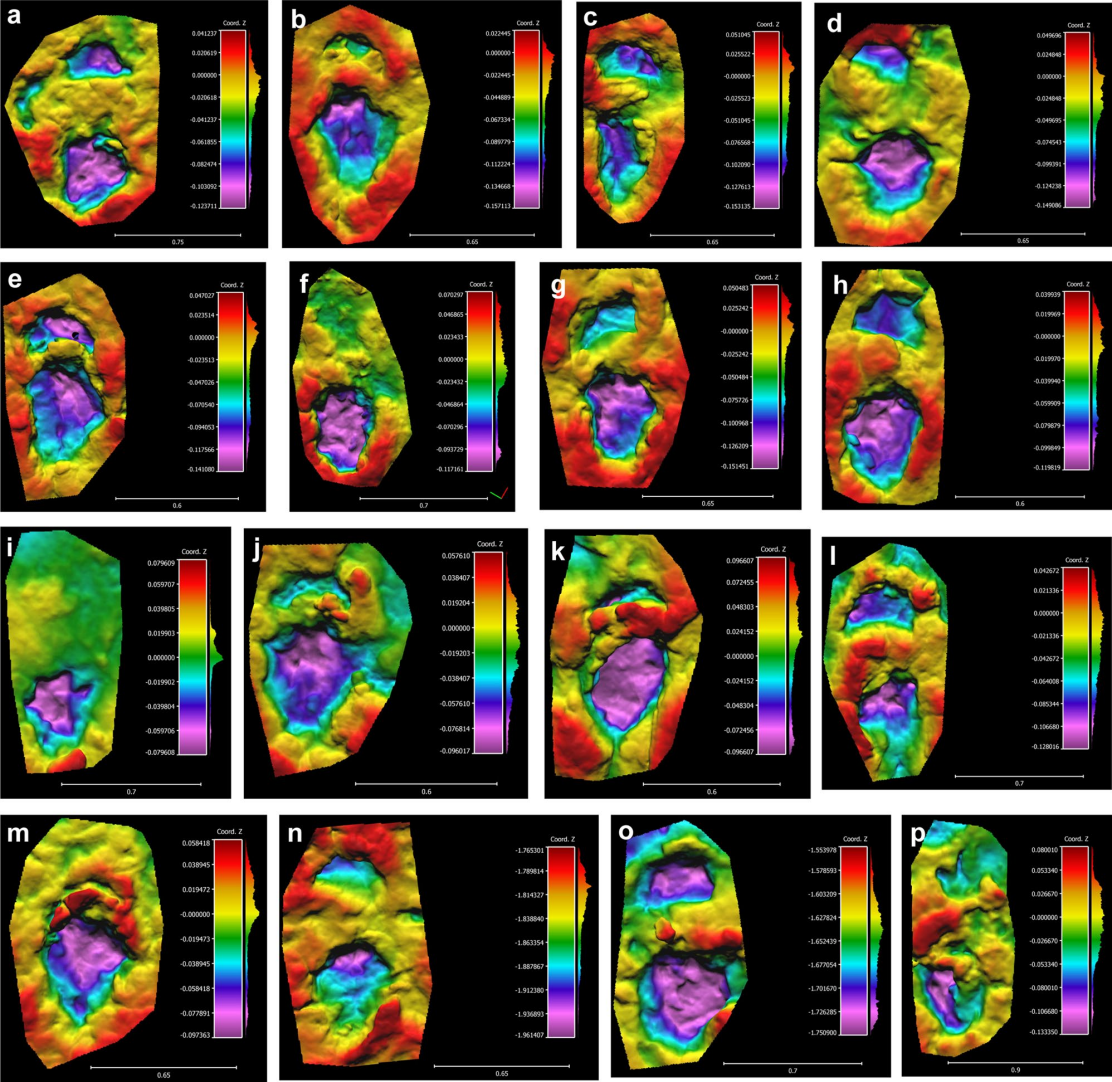
Las Sereas 8 tracksite. Tracks of *Iniestapodus burgensis*. False-color depth maps: (**a**) Pair LS8A,1p/m; (**b**) Pair LS8A,2p/m; (**c**) Pair LS8A,3p/m; (**d**) Pair LS8A,4p/m; (**e**) Pair LS8A,5p/m; (**f**) Pair LS8A,6p/m; (**g**) Pair LS8A,13p/m; (**h**) Pair LS8A,14p/m; (**i**) LS8A,16p; (**j**) Pair LS8A,19p/m; (**k**) Pair LS8A,21p/m; (**l**) Pair LS8A,22p/m; (**m**) Pair LS8A,23p/m; (**n**) Pair LS8A,24p/m; (**o**) Pair LS8A,25p/m; (**p**) Pair LS8A,26p/m. The false-color depth maps were generated with CloudCompare, a free software (http://www.cloudcompare.org/).

**Figure 5:**
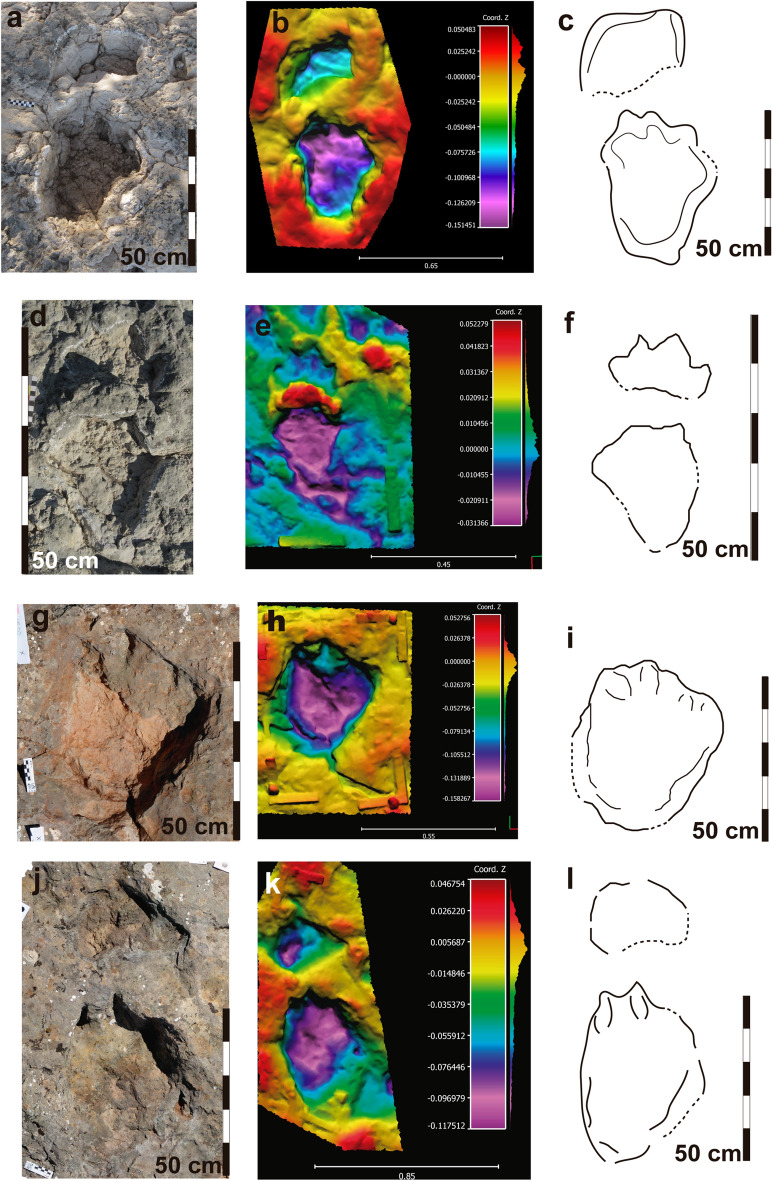
Other tracks of *Iniestapodus burgensis.* Pair LS8A,13p/m: (**a**), (**b**), (**c**). Pair LS8B,2p/m: (**d**), (**e**), (**f**). Pes track LS3,1: (**g**), (**h**), (**i**). Pair LS3A, 1p/m: (**j**), (**k**), (**l**). Left images: photographs (**a);** ortophotos (**d**), (**g**), (**j**). Central images: False-color depth maps (**b**), (**e**), (**h**), (**k**). Right images: Interpretative sketches (**c**), (**f**), (**i**), (**l**). The false color depth maps were generated with CloudCompare, a free software (http://www.cloudcompare.org/).

Manus tracks indicate a morphology that varies from semicircular to crescent-shaped being wider than long (Figs. 2, 3, 4 and 5; Supplementary Table S1). Those of the LS7A and LS7B trackways are the largest ones (Supplementary Tables S2, Table S3). Averaged manus tracks measurements are: LS7A, 29 cm long and 40 cm wide, and LS7B, 28 cm long and 31 cm. The manus tracks of LS7C and LS8A trackways and manus-pes set LS3A, 1p/m are slightly smaller and their mean values are 18 cm long and 28.5 cm wide in LS7C and 23 cm and 39 cm in LS8A (Supplementary Table S3). The manus track LS3A,1m is 18 cm long and 24 cm wide. Finally, the smallest manus tracks are those of LS8B, which measure an average of 11.7 cm long and 19.1 cm wide. The best-preserved manus impressions have three rounded digit impressions oriented anteriorly (digit II, III, and IV) and a small subcircular pollex impression oriented medially (e.g., LS7A, 8 m, 9 m, 10 m; LS8A,1m; Figs. 2b and 3i–n). Several of them are deformed by overprinting of the pes tracks. The holotype manus track is semicircular, measures 29 cm long and 38 cm wide, and has a small and subcircular pollex impression (Supplementary Table S1; Fig. 3b–e). The morphological quality is variable from 0.5 to 2 (holotype has a value of 2).

Pes tracks are subrectangular in shape and the posterior edge is slightly protruding. They reveal four claw impressions occupying the entire width of the anterior zone, with the external claw impression (IV) located laterally and the internal (I) medially (Figs. 3, 4, 5). Claw I impression is in a more backward position than the others, and it is smaller than II, III, and IV that are similar in size (IV a bit smaller than II and III) (Fig. 3a,e). Claw I and II impressions are anteriorly directed, while III and IV are anterolaterally oriented. There are no impressions of a blunt callosity or a claw impression at the distal-lateral end of digit IV that might suggest the presence of a digit V impression. The pes tracks are longer than wide (Supplementary Table S3), and those of LS7A and LS7B are slightly larger than LS7C, LS8A, LS3A,1p, and LS3,1 but within the medium-sized class category. The means pes track measurements are: LS7A, 60 cm long and 43 cm wide; LS7B, 63 cm long and 43 cm wide; LS7C, 55.7 cm long and 33.7 cm wide; LS8A, 54 cm long and 41 cm wide. LS3A,1p/m presents a length–width of 57–34 cm and LS3,1 of 51–39 cm. The smallest pes tracks are those of LS8B (small size class), which measure a mean of 27 cm long and 23 cm wide. LS7 pes tracks are those with the highest morphological quality (some, including the holotype, with > 2 on the morphological quality preservation scale). Pes tracks are generally deeper than the manus tracks (Figs. 3d, 4, 5b,e,k). The holotype pes track is subrectangular in shape and measures 65 cm long and 37 cm wide (Supplementary Table S1; Fig. 3b–e). Within the four claw impressions, the claw I is the smallest one and is medially located whereas claw III is the largest and is laterally rotated. The heteropody is variable from low to medium. For instance, LS8A reveals an heteropody of 1:2.7 and an heteropody index of 38.2% (Supplementary Table S2) whereas LS7A and LS7B trackways have the same values: 1:2.5 and 41% respectively.LS7C, LS8B, and LS3A have an heteropody of 1:3.7, 1:2.8, and 1:4.5, and a heteropody index of 47%, 37%, and 22% respectively.

The trackways LS7A, LS7B, LS7C, and LS8A are sinuous along their lengths and are ~ 7 m, 6 m, 4 m, and 19 m long, respectively (see Torcida Fernández-Baldor et al.^18^ for a complete description of LS7 trackways; Fig. 1a,b). The distances between the pes tracks and the manus tracks vary (Fig. 2f,g), from more than 40 cm (e.g., LS8A, 1p/m) to a partial overlap (e.g., LA8A, 2p/m), probably due to the changes in trackway direction. LS7A comprises ten manus-pes couples. Trackways LS7B and LS7C are incomplete. The former has seven pes tracks and four manus tracks, while the latter presents three pes tracks and four manus tracks. LSA8 trackway preserves 24 pes tracks and 22 manus tracks (Fig. 2a,b). Some of the tracks are missing due to the broken surface or overlapping among some tracks (Fig. 2a,f). The LS7 tracks are generally deeper and have clear outlines (especially pes tracks), while those of LS8 are shallower. The trackway parameters are slightly different between LS7 and LS8 trackways (see Supplementary Table S2, and Torcida Fernández-Baldor et al.^18^ Tables 2 and 3). The pace length, of either the manus or pes, is variable among the trackways and within the same trackway. The same occurs with the stride length, track rotation, pace angulation, and the distance between manus and pes tracks (see Supplementary Table S1 and Torcida Fernández-Baldor et al.^18^, Tables 2 and 3). The trackway gauge is intermediate: the PTR value^35^ of LS7A is 43.9, and 47.6 in LS7B; in LS8A the PTR value is 35.3, which indicates an intermediate/wide-gauge trackway (Supplementary Tables S2, S3).

### Remarks

The ichnotaxonomy of the tracks of obligate quadrupedal dinosaurs is a complex topic. The tracks of sauropodomorphs, thyreophorans, and ceratopsians are represented in this category^19,39,40,51–53^. *Iniestapodus* differs from ceratopsian and thyreophoran (ankylosaurian and stegosaurian) ichnotaxa in the number (four vs three to five), morphology (sharp vs blunt), and orientation (two-digit impressions oriented anteriorly and the other two laterally vs anteriorly) of pes claw impressions^52–58^. Regarding coeval ichnotaxa of other non-sauropod quadrupedal dinosaurs, the thyreophoran ichnogenus *Deltapodus*^56^ has a mesaxonic pes with three functional and anteriorly oriented digits; it differs from the tetradactyl pes of *Iniestapodus* with two anteriorly- and two -oriented digits. *Deltapodus* tracks (*D. ibericus* and *D*. isp) have been described in coeval deposits of the Iberian Range^57,58^, and are different to *Iniestapodus* in manus and pes shape, the tridactyl condition of the pes and, especially, in the blunt claw impressions. *Metatetrapous valdensis*, a thyreophoran ichnotaxon from the Berriasian of Germany, also presents four digital impressions in the pes tracks. It differs from *Iniestapodus* in its tetradactyl manus, a narrower gauge, orientation of the claw impressions, and the heel morphology in the pes^53,54^. Moreover, it is different from some basal sauropodomorph ichnotaxa (e.g., *Otozoum*) typical of the Late Triassic and Early Jurassic, that are tetradactyl, with its well-developed and separated digit I–IV impressions and narrow and elongate heel impressions^59–64^. *Iniestapodus* pes tracks typically always have four distinct claw impressions lacking elongate digit impressions, and its heel imprint is wider.

**Figure 6:**
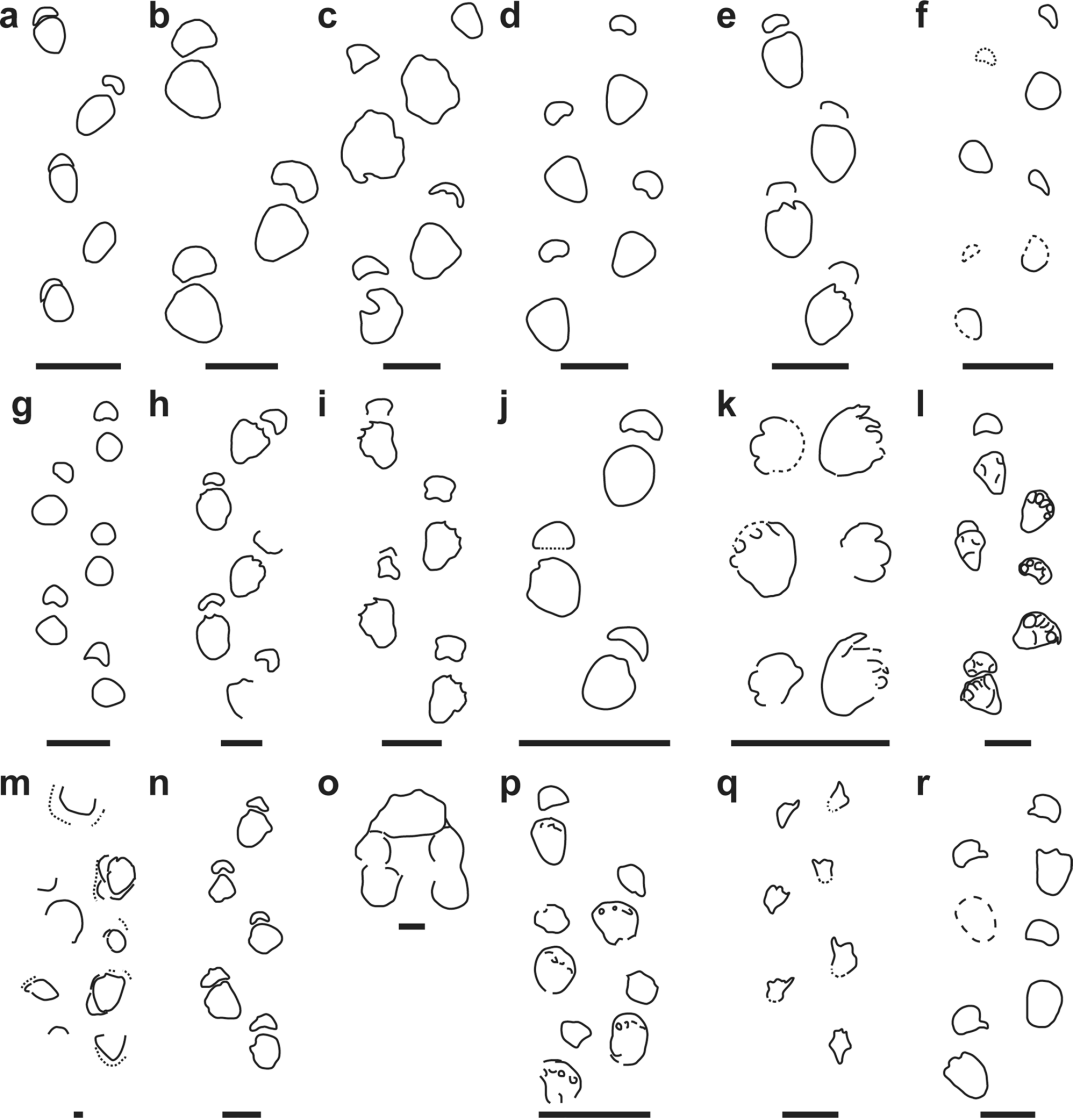
Main sauropod ichnotaxa described in the fossil record. (**a**) *Elephantopoides* Kaever and Lapparent, 1974 (redrawn from^64^). (**b**) *Rotundichnus* Hendricks, 1981 (redrawn from^69^). (**c**) *Gigantosauropus* Mensink and Mertmann, 1984 (redrawn from^70^). (**d**) *Sauropodichnus* Calvo, 1991 (redrawn from^67^). (**e**) *Parabrontopodus*^40^. (**f**) *Titanopodus*^41^. (**g**) *Calorckosauripus* [(redrawn from ^68^]). (**h**) *Breviparopus* Dutuit and Ouazzou, 1980 (redrawn from^71^). (**i**) *Brontopodus birdi*^38^. (**j**) *Brontopodus changlingensis* Chen and Huang, 1993 (redrawn from^72^). (**k**) *Brontopodus pentadactylus*^48^. (**l**) *Brontopodus plagnensis*^73^. (**m**) *Oobardjidama foulkesi*^74^. (**n**) *Occitanopodus gandi*^75^. (**o**) *Titanosaurimanus nana*^76^. (**p**) *Brontopodus oncalensis*^77^. (**q**) *Iguanodonichnus* Casamiquela and Fasola, 1968 (redrawn from^78^). (**r**) *Polyonyx gomesi*^79^. Scale 1 m (5 cm in O).

*Iniestapodus* differs (Fig. 6) from almost all sauropod ichnotaxa because it reveals tetradactyl pes tracks with evidence of four sharp claw impressions. For instance, many of them have less than four claw impressions, or no claw impressions preserved. These include: *Elephantopoides*; *Rotundichnus*; *Gigantosauropus*; *Sauropodichnus*; *Parabrontopodus*; *Titanopodus*; and *Calorckosauripus*^40,41,64–68^ (Fig. 6a–g).

Some sauropod ichnotaxa have pes tracks showing tetradactyl and pentadactyl digit impressions: *Breviparopus*; *Brontopodus* with the ichnospecies: *B. birdi*; *B*. *changlingensis*, *B*. *pentadactylus*; and *B. plagnensis*; *Oobardjidama*; and *Occitanopodus* (Fig. 6h–n)^38,48,73–75,80,81^. The main differences between *Iniestapodus* and all other known sauropod pes tracks are its generally outwardly rotated claw impressions, with a notable decrease in their size from I to IV, with claw IV being very small, and usually blunt, or with no claw trace. Moreover, none of these ichnotaxa preserve a pollex impression in the manus, a key feature in *Iniestapodus*. The sauropod ichnotaxon *Titanosaurimanus*^76^ is known only from a manus track, which has a horseshoe shape, different from the semicircular morphology of the manus of *Iniestapodus* (Fig. 6o). The pes tracks of *Brontopodus oncalensis*^77^ were described as triangular, with four possible claw impressions and the manus tracks as subcircular to pentagonal (Fig. 6p). In a recent visit to the type locality of *B*. *oncalensis* (D.C. and F.T., May 2019), it was not possible to identify the presence of clear claw impressions in the holotype trackway. The track-bearing layer is very eroded, and the tracks are poorly preserved, not reaching a morphological quality higher than 1; thus, this ichnotaxon is herein considered as *nomen dubium* following Marchetti^44^.

On the other hand, the pes tracks of *Iguanodonichnus*^82^, considered as a sauropod ichnotaxon by Moreno and Benton^78^, has four claw impressions, with a prominent claw in digit I and directed forwardly; and claws on digits II, III, and IV are strongly reduced (Fig. 6q). In *Iniestapodus*, the claw I digit impression is the smallest of the pes track.

The ichnotaxon *Polyonyx*^79^, which had been compared to the Las Sereas sauropod tracks in previous work^18^, has a tetradactyl pes that is oval (Fig. 6r). The claw impressions are similar in length, with two claw impressions at an anterior orientation (digits I–II) and the other two (digits III–IV) laterally oriented. Manus tracks have five-digit impressions, with the digit I (pollex) the largest and oriented in a medial to posteromedial direction. *Iniestapodus* shares the orientation of the digit pes impressions with *Polyonyx*, but differs in its general shape (subrectangular vs. oval in shape), size, and the position of pes digit I impressions (smaller than digits II and III, and more backward than the other digit impressions vs. similar size and position than the other digit impressions). Both ichnotaxa are clearly different in manus morphology and especially in the size and orientation of pollex impression (small vs large, and medial-posteromedial vs posterior). Moreover, *Iniestapodus* differs from *Polyonyx* in the heteropody (intermediate to low in the former, and low in the latter) and in trackway gauge (intermediate *Iniestapodus* and wide in *Polyonyx*).

In conclusion, the differences between *Iniestapodus* and the other quadrupedal dinosaur ichnotaxa justify the erection of the new ichnotaxon.

### Trackmaker

In general, an ideal trackmaker identification should reflect similar derived anatomical characteristics (phylogenetic correlation) and the main similarities between the track and the autopod skeleton (phenetic correlation), plus a geographic-temporal distribution (coincidence correlation)^50^. Regarding sauropod pedes and manus, several authors have pointed out an evolutionary process of phalanx reduction, both in the number and proportional size of the phalanges^83–91^. A progressive reduction of the phalanges of the manus is well documented from the sauropodomorphs to the derived titanosaurs, with phalanges vestigial or even absent in the latter^83,88,92–94^. It has been argued that the first pedal phalanx reduction events would occur in the two most lateral digits (IV and V). This process could be due to a functional compensation since the reduction of the lateral digits would occur at the same time as a greater growth of digits I and II^95^. Both processes may have occurred at the origin of the Neosauropoda clade and would also have affected the development of the ungual phalanges^84,90^.

**Figure 7:**
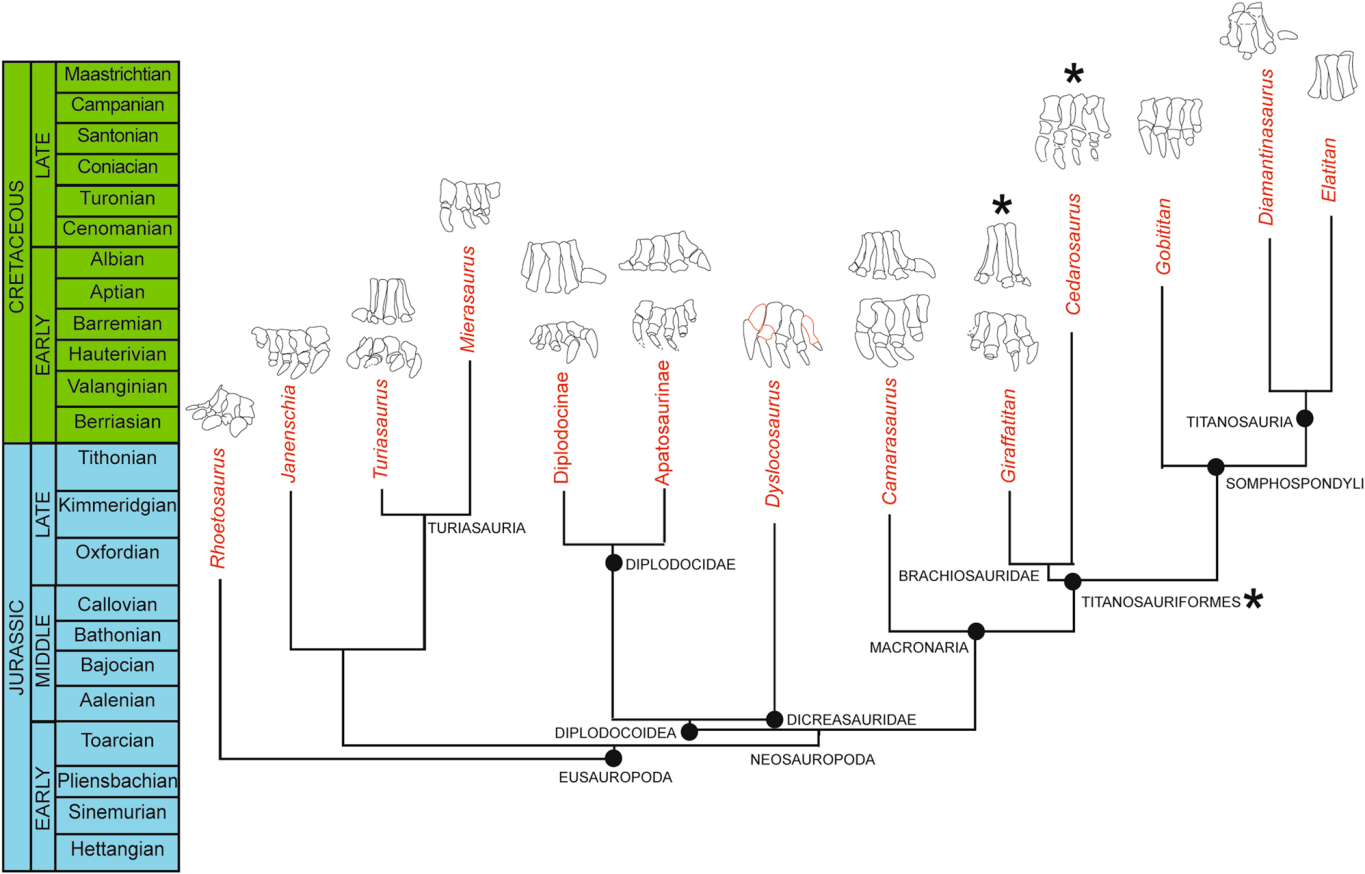
Simplified time-calibrated sauropod phylogenetic tree based on Mannion et al.^91^, with data from Tschopp et al.^87^ and Poropat et al.^88^, showing complete manus and pes of selected Late Jurassic-Cretaceous taxa of each of the main clades. The clade that is proposed as the most probable trackmaker of *Iniestapodus* is marked with asterisks (*). *Rhoetosaurus* (pes redrawn from^96^); *Janenschia* (pes redrawn from^91^); *Turiasaurus* (pes and manus redrawn from^4^); *Mierasaurus* (pes redrawn from^100^); Diplodocinae (pes of *Diplodocus carnegii* redrawn from^101^; manus of an indeterminate diplodocinae following^102^, redrawn from^103^); Apatosaurinae (pes of *Apatosaurus louisae* and manus of *Brontosaurus parvus* redrawn from^104^); Dicreasauridae (pes of *Dyslocosaurus* redrawn from^105^); *Camarasaurus* (pes of *C. lentus* redrawn and reversed from^90^, manus of *Camarasaurus grandis* (or sp) redrawn from^106^ see^94^); *Giraffatitan* (manus redrawn from^107^, pes redrawn from^96^); *Cedarosaurus* (pes redrawn from^108^); *Gobititan* (pes redrawn from^109^); *Diamantisaurus* (manus redrawn from^88^); *Elatitan* (manus redrawn from^110^).

Moreover, a brief review of skeletal feet (Fig. 7) is relevant for the discarding of some sauropod taxa and their corresponding clades. For instance, the *Iniestapodus* pes track morphology is different from those left by basal sauropods, such as *Rhoetosaurus*, which reveals four ungual phalanges, with the ungual of digit I the largest, and decreasing in size until digit IV; the ungual of digit I is located more anteriorly than the others, and the rest of the claws are oriented towards the anterior, lateral or lateroposterior^86,96^. Moreover, the *Iniestapodus* trackmaker differs from basal eusauropods that have three ungual phalanges, which are relatively large, and are anterolaterally oriented with a decreasing size from digit I to III^50,97–99^. In Eusauropoda, the impression of the claw in digit IV would be small (if present)^46^. These features can be seen in some Late Jurassic non-neosauropod eusauropods such as *Janenschia*^91^. Late Jurassic–Early Cretaceous turiasaurids (e.g., *Turiasaurus, Mierasaurus*) also have three ungual phalanges (I–III), and a digit IV with just two phalanges and without a claw; ungual I is more developed than the other two, of which claw III is relatively small^4,100^. Within Neosauropods, pes impressions should be similar, with the main differences being in the number (two, three, or four) and the relative sizes of the pedal claws^46^.

Regarding diplodocoids, the articulated feet MB.R.2370 and MB.R.2371 from the Tithonian of the Tendanguru Beds and the feet of some *Diplodocus* Marsh, 1878 individuals from the Kimmeridgian-Tithonian Morrison Formation, show only two claws on digits I and II^101,107,111^. Moreover, the Late Jurassic *Apatosaurus* Marsh, 1877, and other diplodocine individuals show ungual phalanges on digits I–III, with I and II more developed than the III^107,111^. Digit IV lacks an ungual and is shorter than the other digits^102–104,112^. Within dicraeosaurs, *Dyslocosaurus* McIntosh et al., 1992^105^ preserved a foot with four claws that decrease in size from digit I to IV, and with an especially robust claw on digit I. The Late Jurassic macronarian *Camarasaurus* Cope, 1887 reveals well-developed claws on digits I through III, the claw I being larger than the other two, but the presence of a claw on digit IV is doubtful because the digit IV in *Camarasaurus* was short, with phalanges reduced in size^87,113,114^. Some Late Jurassic–Early Cretaceous non-titanosaurian titanosauriforms have three ungual phalanges, such as *Tastavinsaurus*^115^, *Gobititan*^109^, or an indeterminate titanosauriform from Russia^116^, whereas others, such as *Cedarosaurus*^108,117^, show four ungual phalanges. The pedes of the aforementioned non-titanosaurian titanosauriforms show claws with slightly decreasing sizes, but without large differences between them, particularly claw I with respect to claws II and III.

In summary, there are only two clear reports of neosauropods with four unguals on digits I–IV that fit with the morphological features of the pes tracks of *Iniestapodus:* the Early Cretaceous brachiosaurid *Cedarosaurus*^108,117,118^ and the dicraeosaurid *Dyslocosaurus*^102,105^. Additionally, it should be noted that Jensen^113^ reported a reduced fourth ungual phalanx in a pes of *Camarasaurus*, although other complete feet of this species only show three unguals^87,114^. The former authors^87^ suggested that “…*this underlines that the exact pedal phalangeal formula is still in question in many genera, and that this formula might be inter- or even intraspecifically variable*”. This highlights the difficulties in the identification of sauropod trackmakers based only on pes morphology. Finally, Cretaceous titanosaurs are characterized by a reduction in size or loss of pedal phalanges. They have three ungual phalanges with the digits I and II being generally similar in size, and with digit III being generally smaller or similar in size. Digit IV is much smaller than the other digits and lacks ungual phalanges^46,85,89,119^.

Despite the multi-source data provided by the pedal skeleton record, an analysis of sauropod manus osteology and the osteological record allows us to discard many of the aforementioned clades as potential trackmakers (Fig. 7). The manus morphology of *Iniestapodus* shows a small pollex impression that is key for identifying the sauropod clade. The sauropod fauna currently identified across the Jurassic–Cretaceous transition of the Iberian Peninsula include mainly non-neosauropod eusauropods (including turiasaurs), diplodocoids (some specimens with diplodocine affinities), basal macronarians (non-camarasaurids and camarasaurids), and brachiosaurid titanosauriforms^3,15^. Many of these clades can be discarded from consideration since they have a very large pollex (Fig. 7) that presumably would leave a large impression^51,94,106,114^. Thus, eusauropods (including turiasaurs)^4^, some neosauropod clades such as camarasaurids^87,113^, and diplodocids^103,104,106^ could be discarded as a candidate trackmaker of *Iniestapodus*. Interestingly, the manus morphology of some titanosauriforms, such as the brachiosaurid *Giraffatitan*^120^ has a reduced pollex^107,121^. In general, the most derived titanosauriforms, the titanosaurs, reveal a loss of ungual phalanges in the manus^110,122–124^, with some exceptions (*Diamantinasaurus*^88,93^). Other groups, such as dicraeosaurids or titanosaurs, can be discarded since there is no record of their presence on the Iberian Peninsula or in Europe during this period.

Consequently, the analysis and comparison carried out between the anatomical characteristics preserved in the tracks (small ungual phalanx in the manual digit I, four ungual phalanges in the pes with unguals I and II sub-equal in size), and the sauropod bone record in the Iberian Peninsula suggest that titanosauriforms are the most plausible trackmakers. These data also fit with the predictions provided by Day et al.^125^ and Wright^46^. The former authors indicated that in brachiosaurids (and the most basal titanosaurs) the trackways should show intermediate-gauge and well-developed or reduced manus claw impressions. The presence of titanosauriforms on the Iberian peninsula is well-documented in the Jurassic-Cretaceous transition^3,14,126–128^, including a primitive brachiosaurid in the same deposits of the Rupelo Formation^16^, and thus this group of non-titanosaurian macronarians is the best candidate to have been the trackmaker of *Iniestapodus*. The only group that cannot be completely ruled out is the non-camarasaurid basal macronarians, since neither complete feet nor manus have been found; hence, there are no clear data regarding the trackway type of this sauropod group. Moreover, they co-occur in almost coeval deposits of the Iberian Range (e.g., *Aragosaurus*^7,129^), but with the current data, it is not possible to confidently consider them as plausible trackmakers.

### Paleoecological and paleoenvironmental inferences

**Figure 8:**
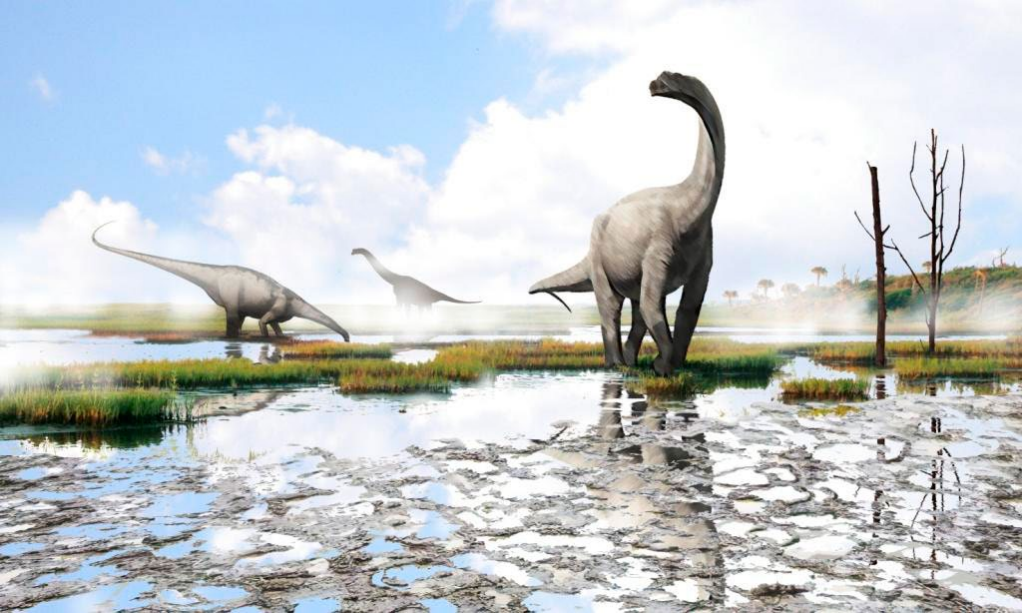
Paleoenvironmental reconstruction of the Las Sereas megatracksite. Illustration made with free software Autodesk SketchBook (https://www.autodesk.com/products/sketchbook/overview).

The Las Sereas tracksite is composed of several outcrops along a 5.6 km exposure of the top of the same horizon of lacustrine limestone strata. It covers an area of approximately 448,000 m^2^. The number of outcrops at Las Sereas is probably greater than the 14 tracksites currently known, because vegetation and debris partially conceal the track-bearing strata, and further prospecting is required^130^. A megatracksite is traditionally considered a regionally extensive track-bearing stratification surface with a lateral extension of hundreds or even thousands of meters^19,131^. Megatracksites are usually related to environments with large lateral extensions, such as coastal plains, tidal flats, or lacustrine settings^19,131–136^. Accordingly, Las Sereas can be considered a lacustrine megatracksite in a coastal wetland. It represents low-gradient lake margins in which seasonal lake level oscillations occurred. During the dry season in these coastal wetlands, the water would evaporate and only remain in the central parts of the lakes, creating a wide, exposed carbonate-mud plain undergoing progressive drying, with perfect conditions for impressing and preserving the tracks^30^ (Fig. 8). This plain would have had concurrent areas with very soft and firm muds resulting in a mixture of deep and shallow, and well and poorly preserved tracks^137^. For instance, LS7 and LS8 are both on the same track-bearing surface and have tracks similar in size, but LS8 shows shallower tracks than LS7. It is possible that the substrate was drier when the LS8 trackway was registered than during the impressions of LS7. Moreover, although all the trackways preserve clear displacement rims, LS7 (see Fig. 3a–e) are more prominent than in LS8 (Figs. 2, 4). Similar lateral changes have been described in present-day tracks from tidal flats^45,138^.

Megatracksites can provide a broad spatial picture of the fauna present in an area^19,131^. The Las Sereas ichnoassociation is similar to those of other Laurasian Jurassic–Cretaceous units, comprising thyreophoran, sauropod, ornithopod, and theropod footprints^18^. It should be noted that almost all the sauropod tracks fall into two size categories: medium-sized (LS7A, LS7B, LS7C, LS8A) and small-sized (LS8B). The presence of tracks with the same shape and much smaller size (LSA8B) suggests that the trackmakers found at Las Sereas represent sauropods of two different ontogenetic stages^137^.

The orientation of the trackways may be evidence of gregarious behavior [e.g.,^1,47^]. Nonetheless, in Las Sereas 7 the three analyzed trackways intersect each other with different directions of displacement (see Supplementary Fig. S2). In the extensive Las Sereas 8 site, the two studied trackways have different directions and are far from each other; in addition, several isolated sauropod tracks are arranged far from the two sauropod tracks of the site. Therefore, the possibility of gregarious behavior of the *Iniestapodus* trackmaker cannot be confidently inferred with the current data. Otherwise, the trackways indicate a random orientation pattern that represents directional or milling behaviour across the site^1,139^. The relative abundance of *Iniestapodus* at the Las Sereas megatracksite could indicate that the trackmaker used these shallow lacustrine/palustrine areas as its preferred habitat, as does the brachiosaurid registered in lower levels of the Rupelo Formation^15^.

## Conclusions

The sauropod tracks studied herein were impressed in three exposures of the Las Sereas megatracksite, where more than 1000 vertebrate tracks, along 5.6 km, have been identified at ~ 14 sites. They are preserved on the upper bedding plane surfaces of lacustrine strata from the upper part of the Rupelo Formation (Berriasian, Cameros Basin). The sauropod tracks belong to a new ichnotaxon *Iniestapodus burgensis*. *Iniestapodus* is characterized by subrectangular tetradactyl pes tracks with short claw I impression, forwardly oriented claw I and II impressions, and slightly rotated and curved laterally claw III and IV impressions. The manus tracks are semicircular to crescent shape, with three broad digit impression oriented posteriorly, and a short digit I impression medially located. Accordingly, non-titanosaurian titanosauriforms have been proposed as possible trackmakers, whereas other coeval quadrupedal dinosaurs (e.g., thyreophorans) are discarded. This group of sauropods, and brachiosaurids, particularly, are present in the Rupelo Formation as well as at other sites from the Jurassic–Cretaceous transition on the Iberian Peninsula. The only sauropod track morphotype present in the Las Sereas tracksite is *Iniestapodus*, which shows different track sizes and trackway orientations in the same outcrop and between outcrops. The data obtained here suggest that the trackmakers utilised shallow lacustrine/palustrine areas as their preferred habitat and that the trackways were produced by solitary individuals of different ontogenetic stages.

## Supplementary Information

Supplementary Information 1.

Supplementary Information 2.

Supplementary Information 3.

Supplementary Information 4.

Supplementary Tables.

## Data Availability

The datasets generated during the current study are available from the corresponding author on reasonable request.
