# Supplementary material for: Enigmatic tracks of solitary sauropods roaming an extensive lacustrine megatracksite in Iberia
Source: Sci Rep. 2021 Aug 20;11:16939. doi: 10.1038/s41598-021-95675-3 (PMC8379178; doi:10.1038/s41598-021-95675-3)
Supplement: Supplementary file 1 — Supplementary Information 1. [file 41598_2021_95675_MOESM1_ESM.docx]

**Supplementary figure 1 caption.** Aerial view of the Las Sereas 7 and Las Sereas 8 sites (marked by red arrows). The red lines indicate the extent of each site and the connection between them. Photograph were made for Fidel Torcida Fernández-Baldor.
