# Supplementary figures and images for: Enigmatic tracks of solitary sauropods roaming an extensive lacustrine megatracksite in Iberia
Source: Sci Rep. 2021 Aug 20;11:16939. doi: 10.1038/s41598-021-95675-3 (PMC8379178; doi:10.1038/s41598-021-95675-3)

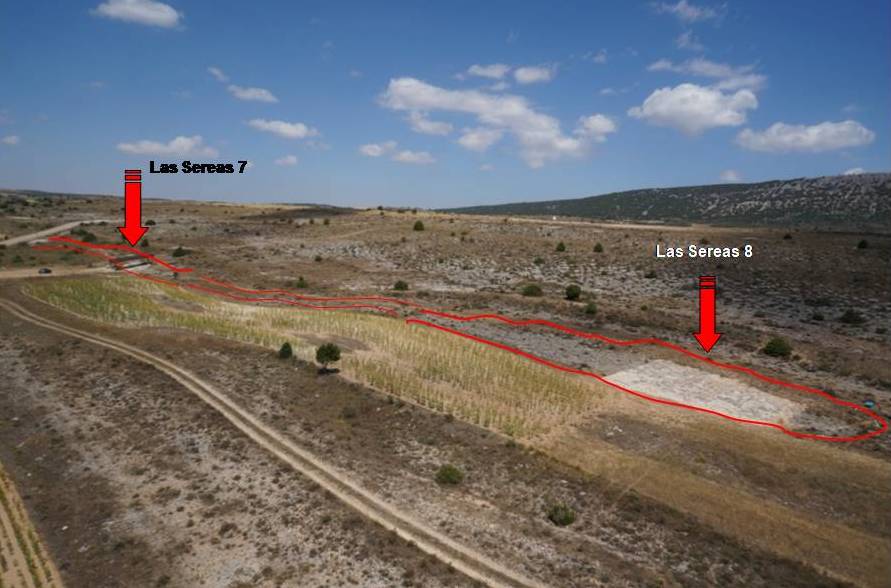

Supplement: Supplementary file 2 — Supplementary Information 2. [file 41598_2021_95675_MOESM2_ESM.jpg]
