# Supplementary material for: Enigmatic tracks of solitary sauropods roaming an extensive lacustrine megatracksite in Iberia
Source: Sci Rep. 2021 Aug 20;11:16939. doi: 10.1038/s41598-021-95675-3 (PMC8379178; doi:10.1038/s41598-021-95675-3)
Supplement: Supplementary file 3 — Supplementary Information 3. [file 41598_2021_95675_MOESM3_ESM.docx]

**Supplementary figure 2 caption.** Rose plot showing the orientation (multideractional pattern) of *Iniestapodus burgensis* trackways (LS7A, LS7B, LS7C, LS8A), LS8B and LS8C) in the different outcrops of Las Sereas megatracksite. Diagram carried out with the software GeoRose (<http://www.youngtechnology.com/download/georose>) trackways LS7C and LS8 are turning trackways a mean value of the different direction has been used in the diagram.
