# Supplementary material for: Enigmatic tracks of solitary sauropods roaming an extensive lacustrine megatracksite in Iberia
Source: Sci Rep. 2021 Aug 20;11:16939. doi: 10.1038/s41598-021-95675-3 (PMC8379178; doi:10.1038/s41598-021-95675-3)
Supplement: Supplementary file 4 — Supplementary Information 4. [file 41598_2021_95675_MOESM4_ESM.pdf]

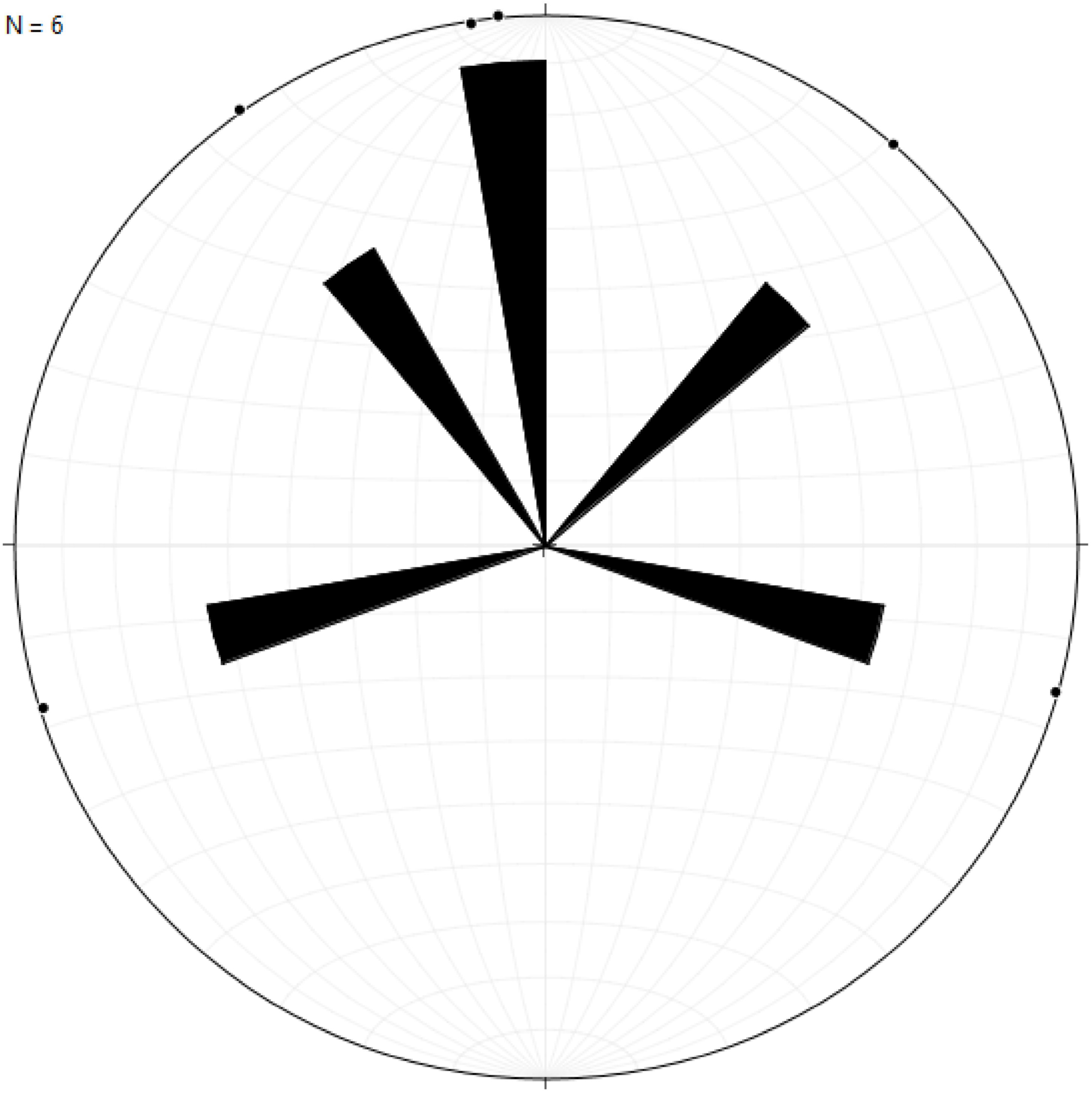

----- Rose diagram/Circular Histogram | 12/03/2021 at 10:34-----
Lines data set: Untitled Lines
Max value = 28.57143% between 171° and 180°
Mean Vec = 354.6° ± 59.4°; Average Length = 0.2575
Circular Variance = 0.7425; kappa = 0.5165
[Krumbein's axial mean; uncertainty is 1 standard error,
for 95% confidence level multiply by 1.96]

----- Rose diagram/Circular Histogram | 12/03/2021 at 10:40-----
Lines data set: Untitled Lines
Max value = 33.33333% between 171° and 180°
Mean Vec = 348.1° ± 117.4°; Average Length = 0.1404
Circular Variance = 0.8596; kappa = 0.2828
[Krumbein's axial mean; uncertainty is 1 standard error,
for 95% confidence level multiply by 1.96]
