# Supplementary material for: Enigmatic tracks of solitary sauropods roaming an extensive lacustrine megatracksite in Iberia
Source: Sci Rep. 2021 Aug 20;11:16939. doi: 10.1038/s41598-021-95675-3 (PMC8379178; doi:10.1038/s41598-021-95675-3)
Supplement: Supplementary file 5 — Supplementary Tables. [file 41598_2021_95675_MOESM5_ESM.docx]

| **Trackway LS8 A** | **FL (p / m)** | **FW (p / m)** | **PL (p / m)** | **SL (p / m)** | **iTW (p / m)** | **eTW (p / m)** | **FR (p / m)** | **ANG (p / m)** | **Dm-p** | **Gad** |
| --- | --- | --- | --- | --- | --- | --- | --- | --- | --- | --- |
| **LS8 A, 1 p/m** | 52 / 25 | 49 / 48 |  |  |  |  |  |  | 68 | 122 |
| **LS8 A, 2 p/m** | 62 / (20)* | 39 / 36 | 107 / 119 | 151 / 139 | 22 / 56 | 109 / 134 | 1º / 13º | 97º / 74º | 43 | 75 |
| **LS8 A, 3 p/m** | 54 / 22 | 27 / 39 | 92 / 112 | 134 / 148 | 27 / 50 | 111 / 122 | 22º / 20º | 91º / 84º | 51 | 120 |
| **LS8 A, 4 p/m** | 54 / (18)* | 42 / (32)* | 94 / 111 | 150 / 143 | 25 / 43 | 116 / 116 | 30º / 22º | 94º / 83º | 58 | 120 |
| **LS8 A, 5 p/m** | 54 / 16 | 42 / 33 | 110 / 104 | 114 / -- | 25 / 42 | 119 / -- | 17º / 27º | 72º / -- | 41 | 118 |
| **LS8 A, 6 p/m** | 49 / - | 36 / - | 78 / -- | 100 / 141 | 14 / 47 | 105 / -- | 1º / -- | 81º / -- | 80 | 120 |
| **LS8 A, 7 p/m** | 50 / (23)* | 44 / (31)* | 74 / -- | 111 / -- | 10 / 34 | -- / -- | 7º / 24º | 90º / -- | -- | 122 |
| **LS8 A, 8 p/m** | -- / -- | -- / -- | 81 / 92 | 133 / 108 | 11 / 40 | -- / -- | -- / -- | 99º / 73º | 54 | 115 |
| **LS8 A, 9 p/m** | -- / -- | -- / 34 | 96 / 91 | -- / -- | 14 / -- | -- / -- | -- / -- | -- / -- | 47 | 123 |
| **LS8 A, 10 p/m** | -- / -- | -- / -- | -- / -- | -- / 145 | -- | -- / -- | -- / -- | -- / -- | -- | 131 |
| **LS8 A, 11 p/m** | -- / -- | -- / 44 | -- / -- | -- / -- | -- | -- / -- | -- / -- | -- / -- | -- | 139 |
| **LS8 A, 12 p/m** | -- / 22 | -- / 35 | -- / 128 | -- / 145 | -- / 54 | -- / 132 | -- / -- | -- / 73º | -- | 143 |
| **LS8 A, 13 p/m** | 58 / 18 | 39 / 39 | -- / 113 | 128 / 152 | -- / 48 | -- / 127 | 8º / 13º | -- / 80º | 49 | 120 |
| **LS8 A, 14 p/m** | 58 / 24 | 41 / 37 | 99 / 122 | 116 / 140 | 32 / 52 | 118 / 132 | 1º / 25º | 81º / 72º | 56 |  |
| **LS8 A, 15 p/m** | 53 / (11)* | 43 / 35 | 99 / 114 | -- / | 34 / | 118 / | -- / -- | 77º / -- | 57 |  |
| **LS8 A, 16 p/m** | 48 / -- | 39 / -- | 89 | -- | -- | -- / -- | -- / -- | -- / -- | -- |  |
| **LS8 A, 17 p/m** | -- | -- | -- | -- | -- | -- / -- | -- / -- | -- / -- | -- |  |
| **LS8 A, 18 p/m** | -- | -- | -- | -- | -- | -- / -- | -- / -- | -- / -- | -- |  |
| **LS8 A, 19 p/m** | (54)* / (12)* | 48 / 36 | -- | -- | -- | -- / -- | -- / -- | -- / -- | 38 |  |
| **LS8 A, 20 p/m** | -- / (26)* | -- / (33)* | 111 / 136 | 157 / 153 | 31 / 44 | 122 / 121 | 0º / 14º | 91º / 82º | 57 |  |
| **LS8 A, 21 p/m** | (61)* / -- | 44 / 36 | 109 / 92 | 143 / 146 | 31 / 42 | 119 / 123 | - 2º / 7º | 87º / 79º | 35 |  |
| **LS8 A, 22 p/m** | -- / -- | 42/ 46 | 96 / 132 | 146 / 155 | 26 / 45 | 115 / 132 | 10º / 14º | 91º / 81º | 62 |  |
| **LS8 A, 23 p/m** | 59 / -- | 42 / 40 | 107 / 106 | 163 / 161 | 32 / 46 | 123 / 128 | - 9º / 16º | 90º / 83º | 40 |  |
| **LS8 A, 24 p/m** | 60 / 29 | 44 / 39 | 121 / 133 | 164 / 181 | 29 / 52 | 124 / 134 | - 1º / 6º | 91º / 86º | 59 |  |
| **LS8 A, 25 p/m** | 56 / 27 | 52 / 44 | 107 / 129 | 200 / 169 | 23 / 59 | 126 / 145 | - 12º / 36º | 107º / 78º | 65 |  |
| **LS8 A, 26 p/m** | -- / -- | 52 / -- | 141 / 139 | 166 / 154 | 24 / 51 | 135 / 143 | 3º / -11º | 89º / 73º | 30 |  |
| **LS8 A, 27 p/m** | -- / -- | 51 / 42 | 90 / 118 |  |  |  |  |  | 50 | **124** |
| ***AVERAGE*** | **55 / 23 Except *** | **41 / 39 Except *** | **106 / 116** | **142 / 149** | **24 / 45** | **119 / 130** | **5º / 16º** | **89º / 79º** | **52** | **52** |

**Supplementary Table S1**. Measurements and ratios of sauropod trackway LS8A from La Sereas 8 (Burgos, Spain). Abbreviations: Footprint length (FL), footprint width (FW), pace length (PL), stride length (SL), inner and outer trackway width (iTW–eTW), footprint rotation (FR), pace angulation (ANG), distance manus-pes (Dm-p); glenoacetabular distance (Gap).

| **Trackway LS8 A** | **H** | **HI** | **PTR** | **MTR** | **WAP/WAM** | **WAP/PL** | **WAM/PL** | **H** | **v (Km / h)** |
| --- | --- | --- | --- | --- | --- | --- | --- | --- | --- |
| **LS8 A, 1 p/m** | 1 : 2,1 | 48 |  |  |  |  |  | 207,6 |  |
| **LS8 A, 2 p/m** | 1: 3,3 | 30 | 35,6 | 26,6 | 66 / 92 | 0,62 | 0,77 | 246,4 | 1,95 |
| **LS8 A, 3 p/m** | 1: 1,7 | 58 | 23,9 | 31,6 | 65 / 82 | 0,7 | 0,73 | 215,2 | 1,87 |
| **LS8 A, 4 p/m** | 1: 4* | 25* | 35,5 | 27,2* | 69 / 81 | 0,73 | 0,72 | 217,6 | 2,2 |
| **LS8 A, 5 p/m** | 1: 4,3 | 23 | 34,5 | -- | 71 / -- | 0,65 | -- | 214 | 1,4 |
| **LS8 A, 6 p/m** | -- | -- | -- | -- | 57 -- | 0,73 | -- | 195,6 | 1,3 |
| **LS8 A, 7 p/m** | 1: 3 | 33 | -- | -- | 54 -- | 0,73 | -- | 198,8 | 2,4 |
| **LS8 A, 8 p/m** | -- | -- | -- | -- | 58 / 73 | 0,72 | 0,79 | -- | -- |
| **LS8 A, 9 p/m** | -- | -- | -- | -- | -- | -- |  | -- | -- |
| **LS8 A, 10 p/m** | -- | -- | -- | -- | -- | -- |  | -- | -- |
| **LS8 A, 11 p/m** | -- | -- | -- | -- | -- | -- |  | -- | -- |
| **LS8 A, 12 p/m** | -- | -- | -- | 26,4 | -- / 96 | -- | 0,75 | -- | -- |
| **LS8 A, 13 p/m** | 1: 3,2 | 31 | -- | 30,8 | 74 / 91 | -- | 0,81 | 233,6 | 1,6 |
| **LS8 A, 14 p/m** | 1: 2,7 | 37 | 34,7 | 27,7 | 72 / 96 | 0,73 | 0,79 | 231,2 | 1,4 |
| **LS8 A, 15 p/m** | 1: 5,8* | 17 | 36,3 | -- | -- | -- |  | 210,8 | -- |
| **LS8 A, 16 p/m** | -- | -- | -- | -- | -- | -- |  | 193,2 | -- |
| **LS8 A, 17 p/m** | -- | -- | -- | -- | -- | -- |  | -- | -- |
| **LS8 A, 18 p/m** | -- | -- | -- | -- | -- | -- |  | -- | -- |
| **LS8 A, 19 p/m** | 1: 6,3* | 16* | -- | -- | -- | -- |  | 214,4* | -- |
| **LS8 A, 20 p/m** | -- | -- | -- | 27* | 77 / 87 | 0,69 | 0,64 | -- | -- |
| **LS8 A, 21 p/m** | -- | -- | 36,9 | 28,9 | 74 / 85 | 0,68 | 0,92 | 243,6* | 1,8 |
| **LS8 A, 22 p/m** | -- | -- | 36,6 | 34,5 | 70 / 92 | 0,73 | 0,7 | -- | -- |
| **LS8 A, 23 p/m** | -- | -- | 33,9 | 31,3 | 81 / 89 | 0,76 | 0,84 | 234,4 | 2,4 |
| **LS8 A, 24 p/m** | 1: 2,3 | 43 | 35,5 | 29,3 | 79 / 95 | 0,65 | 0,71 | 240 | 2,3 |
| **LS8 A, 25 p/m** | 1: 2,4 | 41 | 41,3 | 30,3 | 72 / 103 | 0,67 | 0,8 | 223,6 | 3,5 |
| **LS8 A, 26 p/m** | -- | -- | 38,4 | -- | 76 / 104 | 0,54 | 0,75 | -- | -- |
| **LS8 A, 27 p/m** | -- | -- |  |  |  |  |  | -- |  |
| ***AVERAGE*** | **1: 2,7 Except *** | **38,2 Except *** | **35,3** | **29,7 Except *** | **70 / 90** | **0,69** | **0,77** | **219 Except *** | **2** |

**Supplementary Table S2**. Measurements and ratios of sauropod trackway LS8A from La Sereas 8 (Burgos, Spain). Abbreviations: heteropody index *sensu*  Lockley et al., 1994 (H); heteropody index *sensu* González Riga and Calvo, 2009 (HI); trackway ratio pes (PTR manus trackway ratio (MTR); pes width of the angulation pattern (WAP); manus width of the angulation pattern (WAM); hip height (h) and speed value by the Alexander's (1976) formula (v).

| **Trackway, tracks** | **FL (p / m)** | **FW (p / m)** | **PL (p / m)** | **WAP** | **WAM** | | **WAP / WAM ratio** | **e TW (p / m)** | **PTR / MTR** | **WAP / PL ratio** | **WAM / PL ratio** |  |
| --- | --- | --- | --- | --- | --- | --- | --- | --- | --- | --- | --- | --- |
| **LS8 A** | 54 / 23 | 41 / 39 | 106 / 116 | 70 | 90 | | 0,78 | 119 / 130 | 35,3 / 29,7 | 0,69 | 0,77 |  |
| **LS7 A** | 60 / 29 | 43 / 40 | 86 / 112 | 60 | 90 | | 0,67 | 106 / 115 | 43,9 / 25,8 | 0,69 | 0,8 |  |
| **LS7 B** | 63 / 28 | 43 / 31 | 94 / 109 | 56 | 75 | | 0,75 | 104 / 111 | 47,6 / 31,9 | 0,59 | 0,69 |  |
| **LS7 C** | 55,7 / 18 | 33,7 / 28,5 |  |  |  | |  |  |  |  |  |  |
| **LS8B** | 26,8 / 11,7 | 22,9 / 19,1 |  |  |  | |  |  |  |  |  |  |
| **LS3A (pair pes-manus)** | 56,7 / 18 | 34,4 / 24 |  |  |  | |  |  |  |  |  |  |
| **MEDIA** | **52,7 / 16,8** | **36,3 / 30,3** | **1,4 / 0,74** |  | |  |  |  |  |  |  |  |

**Supplementary Table S3.** Measures and ratios compared of tracks and trackways of Las Sereas 3, 7 y 8. Abbreviations as in Supplementary Table S1.
